# Use of Complementary and Alternative Medicine Among Patients With Diabetes Mellitus: A Cross-Sectional Study

**DOI:** 10.7759/cureus.69288

**Published:** 2024-09-12

**Authors:** Talal Almalki, Abdulrahman G Almalki, Nouf A Alqarni, Rahaf Alsudani, Teaf A Althobaiti, Raghad E Alzahrani

**Affiliations:** 1 Diabetes and Endocrinology, Endocrine and Diabetes Center, King Abdul Aziz Specialist Hospital, Taif Health Cluster, Taif, SAU; 2 Family and Community Medicine, Taif University, Taif, SAU; 3 College of Medicine, Taif University, Taif, SAU

**Keywords:** complementary and alternative medicine, diabetes, pattern, prevalence, saudi arabia

## Abstract

Background

Diabetes mellitus is one of the causes to use complementary and alternative medicine (CAM), which can be classified into nutritional, psychological and physical. The study’s objective was to estimate the prevalence and pattern of using CAM among patients with diabetes mellitus who followed the endocrine and diabetes center in Taif.

Materials and methods

A cross-sectional study was carried out in Taif city from 1 June to 31 July 2023 among diabetic patients aged 14 years and above who followed the endocrine and diabetes center. A valid, reliable, English questionnaire was used in data collection. It was composed of three parts: demographic data, diabetes mellitus history and use of CAM. Patients visited the diabetic center during the study period for their regular appointments were selected randomly and interviewed by trained interns and medical students. By using the Raosoft calculator, the minimal sample size was 361. The chi-square test and independent two-sample t-test were used to investigate the association between categorical variables and continuous numerical variables, respectively. Multivariate logistic regression analysis was performed to control the confounding effect.

Results

A total of 361 patients were included. Their age ranged between 14 and 84 (51.2 ± 16.9 years). Females represented 57.6% of them. Overall, more than a quarter (28.3%) reported using CAM, while 22.4% used CAM for treating diabetes in the last 12 months. The majority of patients (97.1%) used herbs, mainly cinnamon (48.5%), fenugreek/helba (31.3%) and rosemary (20.2%). Their main source of information was family and friends (64.7%). A history of improvement of blood sugar reading with CAM was reported by 61.8%. Multivariate logistic regression analysis revealed that females were at doubled likelihood to use CAM compared to males (Adjusted odds ratio “AOR” = 2.40; 95% confidence interval “CI”: 1.27-4.52, p=0.007). Compared to never-smokers, ex-smokers were more likely to use CAM (AOR=2.87; 95% CI: 1.28-6.43, p=0.010).

Conclusion

The use of CAM, particularly herbs, to treat diabetes is a relatively common practice among Saudi patients. However, the history of informing treating physicians about CAM was reported by a minority of patients.

## Introduction

Diabetes mellitus (DM) has been classified into type 1, type 2, gestational diabetes mellitus and other specific types [1]. Type 2 diabetes is one of the commonest chronic illnesses and its prevalence worldwide has increased from 151 to 463 million from 2000 to 2019 and this number is expected to reach 700 million by the year 2045 [2]. In Saudi Arabia, it has been estimated by the International Diabetes Federation in February 2022 that the prevalence of diabetes reached 17.7% [3]. According to a Saudi health information survey, the total prevalence of diabetes was 14.8% for males and 11.7% for females in 2013 [4].

The United States (US) National Center of Complementary and Integrative Health (NCCIH) defines complementary and alternative medicine (CAM) as a non-mainstream practice that is used together with conventional medicine and “alternative” medicine as a non-mainstream practice replacing conventional medicines [5]. The integration of complementary therapies and conventional treatments known as “integrative medicine” emphasizes safety and evidence while combining the best aspects of both in a more meaningful way [5].

Chronic diseases were one of the causes of the use of complementary and alternative medicine as it was found that 35.5% of participants using CAM were diabetic [6]. Complementary approaches can be classified into nutritional (e.g., special diets, dietary supplements, herbs, and probiotics), psychological (e.g., mindfulness) and physical (e.g., massage, spinal manipulation) [6].

The motivation to conduct the study was our observations of a considerable number of patients who were asking about the use of some herbs and its effect in managing their diabetes in addition to others who expressed their experience using some herbs for that purpose. The primary aim of the study was to study the prevalence and pattern of using complementary and alternative medicine among diabetic patients.

## Materials and methods

Study design

This study was a cross-sectional study carried out in Taif city, which is located in the Makkah Province of Saudi Arabia at an elevation of 1,879 m on the slopes of Al-Sarawat mountains, 2 hours drive from Jeddah. It has a population of around 1 million.

King Abdul Aziz Specialist Hospital is a tertiary hospital with more than 500 beds and has specialized centers like endocrine and diabetes center, cardiac center and oncology center. The endocrine and diabetes center has more than 25000 files of patients and an average of 2900 diabetic patients come every month for their diabetes.

Study criteria

Any male or female patient of the age of 14 years and above with a history of diabetes who visited the diabetic center (King Abdul Aziz Specialist Hospital) during the study period (from 1 June to 31 July 2023) and was willing to give informed consent and participate in the study was eligible to be included in the study.

Sample size calculation

Assuming 50% as the prevalence of using complementary and alternative medicine among diabetic patients, 95% as confidence interval, 80% power and 5% for precision, the minimal sample size was 361.

Assessments

A valid, reliable, English questionnaire used in another research was utilized in data collection, and consent was taken from the principal author [7]. The questionnaire was composed of three parts: demographic data, diabetes mellitus history, and complementary and alternative medicine.

The first part contained questions regarding age, gender, education, employment and marital status while the second part contained questions regarding the diabetes type, duration, type of medications used, complications, comorbidities, smoking status and the last HbA1c. The last part contained questions regarding the use of complementary and alternative medicine in the last 12 months, type, duration, effect on blood glucose, and if any change was made in the management plan by the treating physician.

Procedure

A simple random technique was used to enroll patients visiting the diabetic center during the study period (1 June to 31 July 2023). Patients who met the inclusion criteria and agreed to participate in the study were interviewed by trained interns and medical students using the research tool after the end of their appointments.

Ethical considerations

Approval of the Institutional Review Board (IRB), Directorate of Health Affairs in Taif was taken. In addition, the study’s aim and objectives were explained to each participant and informed consent was signed. Patients were assured of the confidentiality of their information which would only be used strictly for research purposes. Candidates were free to not provide any information they were not comfortable to disclose.

Statistical analysis

Categorical variables were described as frequency and percentages whereas continuous numerical variables were described as arithmetic mean and standard deviation (SD). The chi-square test was used to investigate the association between two categorical variables whereas independent two-sample t-test was used to compare the arithmetic mean of a continuous numerical variable between two different groups. Univariate and multivariate logistic regression analysis was performed to control for the confounding effect and its results were expressed as unadjusted and adjusted odds ratios (AOR) and their 95% confidence interval (CI). P-values of less than 0.05 were considered statistically significant. Data entry and analysis were done using the Statistical Package for Social Sciences (SPSS) software, version 28 (IBM Corp., Armonk, NY, USA).

## Results

Demographic characteristics

A total of 361 patients were included in the study. Their demographic characteristics are summarized in Table 1. Their age ranged between 14 and 84 years, with an arithmetic mean of 51.2 and standard deviation of 16.9 years. Females represented 57.6% of them. Also, two-thirds (65.7%) were married and 51.8% were unemployed. More than a quarter (28.8%) were university graduates/postgraduates. The prevalence of current and ex-smokers was 12.5% and 12.2% among diabetic patients, respectively.

**Table 1:** Demographic characteristics of the participants (n=361) SD: Standard deviation

Variables		Frequency (%)
Gender	Male	153 (42.4)
	Female	208 (57.6)
Marital status	Single	74 (20.5)
	Married	237 (65.7)
	Divorced	12 (3.3)
	Widowed	38 (10.5)
Employment status	Unemployed	187 (51.8)
	Employed	58 (16.1)
	Student	20 (5.5)
	Retired	96 (26.6)
Educational level	Illiterate	83 (23.0)
	Primary-intermediate schools	95 (26.3)
	Secondary school	79 (21.9)
	University/postgraduate	104 (28.8)
Smoking status	Non-smoker	272 (75.3)
	Current smoker	45 (12.5)
	Ex-smoker	44 (12.2)

Diabetes-related characteristics

Most of the patients (71.7%) had type 2 diabetes. The duration of diabetes exceeded 15 years among 39% of patients. Oral hypoglycemics and insulin were used in the treatment of 53.7% and 69% of patients, respectively. Most of the patients (60.7%) reported diabetic complications, mainly retinopathy (33.2%), neuropathy (31.9%) and diabetic foot (26.6%). Also, most of them (70.1%) had comorbid diseases, mainly hypertension (47.6%) and dyslipidemia (41%). Almost two-thirds of patients (67%) had uncontrolled glycemic level (Table 2).

**Table 2:** Diabetes-related characteristics of the participants (n=361) *Not mutually exclusive (i.e., sum exceeded 100%) HbA1c: Glycated hemoglobin

Variables		Frequency (%)
Type of diabetes	Type 1	102 (28.3)
	Type 2	259 (71.7)
Duration of diabetes in years	≤5	79 (21.9)
	6-10	78 (21.6)
	11-15	63 (17.5)
	>15	141 (39.0)
Diabetic medications*	Oral hypoglycemics	194 (53.7)
	Injectable hypoglycemic agents	43 (11.9)
	Insulin	249 (69.0)
Diabetic complications*	Never	142 (39.3)
	Neuropathy	115 (31.9)
	Retinopathy	120 (33.2)
	Nephropathy	41 (11.4)
	Cardiovascular diseases	41 (11.4)
	Diabetic foot	96 (26.6)
Comorbidities*	No	108 (29.9)
	Hypertension	172 (47.6)
	Heart diseases	40 (11.1)
	Thyroid disorders	82 (22.7)
	Dyslipidemia	148 (41.0)
	Others	18 (5.0)
HbA1c% (n=351)	≤7	116 (33.0)
	>7	235 (67.0)

Use of complementary and alternative medicine

Overall, more than a quarter of patients (28.3%) reported using of CAM, while 22.4% used CAM for treating diabetes in the last 12 months.

The pattern of using CAM is fully described in Table 3. The majority of patients, who reported using CAM (97.1%) used herbs, mainly cinnamon (48.5%), fenugreek/helba (31.3%) and rosemary (20.2%). Most of the patients (65.7%) used CAM for a period ranging between more than one month and one year. About 40.2% of them used CAM daily. Their main source of information about CAM was family and friends (64.7%), followed by media and advertisements (24.5%). Histories of improvement of blood sugar reading with CAM and informing treating physicians about CAM were reported by 61.8% and 11.8% of patients, respectively. While patients were on CAM, the majority of them (90.2%) reported that they or their physicians did not make changes in prescription. Regarding the reason for taking CAM, cure of the disease and relief of the disease’s symptoms were mentioned by 41.3% and 33.3% of patients, respectively.

**Table 3:** Pattern of complementary and alternative medicine use among diabetic patients (N=102) *Not mutually exclusive (i.e., sum exceeded 100%) †Cupping is a technique of placing vacuum cups with blood drawn from the surface of the skin by making small surface incisions.

Variables		Frequency (%)
Type of CAM therapies tried for diabetes*	Herbs	99 (97.1)
	Vitamins	3 (2.9)
	Minerals	1 (1.0)
	Roquia (Quran)	3 (2.9)
	Relaxation	1 (1.0)
	Cupping†	4 (3.9)
Type of herbs used for diabetes (n=99)*	Cinnamon	48 (48.5)
	Myrrh	8 (8.1)
	Yansoon	6 (6.1)
	Rosemary	20 (20.2)
	Marjoram	10 (10.1)
	Fenugreek/Helba	31 (31.3)
	Black cumin	6 (6.1)
	Shamer	3 (3.0)
	Barley	6 (6.1)
	Asafetida	3 (3.0)
	Olive/olive leaves	3 (3.0)
	Green tea	2 (2.0)
	Moringa	3 (3.0)
	Ginger	3 (3.0)
	Garlic	2 (2.0)
	Others	21 (21.2)
Duration of using CAM	≤1 month	29 (28.4)
	>1 month-1 year	67 (65.7)
	>1 year	6 (5.9)
Frequency of using CAM	One month at the beginning of diagnosis	3 (2.9)
	Daily	41 (40.2)
	2-4 times/week	33 (32.4)
	Once per week	14 (13.7)
	Monthly	11 (10.8)
Source of information about CAM	Health professionals	6 (5.9)
	Family and friends	66 (64.7)
	Media and advertisements	25 (24.5)
	CAM practitioners	4 (4.9)
History of improvement of blood sugar reading with CAM	No	39 (38.2)
	Yes	63 (61.8)
History of informing treating physicians about CAM	No	90 (88.2)
	Yes	12 (11.8)
While you are on CAM, did you or your physician make changes in the prescription?	No changes	92 (90.2)
	Alternation of time of use	2 (2.0)
	Decreasing the dose	4 (3.9)
	Stopping one or all agents	4 (3.9)
Reason for taking CAM	Cure the disease	42 (41.3)
	Slow down the progression of the disease	18 (17.6)
	Relieve symptoms of the disease	34 (33.3)
	Reduce side effects of medication	8 (7.8)

Factors associated with CAM intake

Demographic Factors

Female patients were more likely to use CAM than male patients (32.7% vs. 22.2%), p=0.029. Other factors (patients’ age, marital status, employment status and educational level) were not significantly associated with CAM use (Table 4).

**Table 4:** Demographic factors associated with complementary and alternative medicine intake among diabetic patients *Chi-square test †Independent two-sample t-test

Variables		Complementary and alternative medicine use		p-value
		No	Yes	
		N=259	N=102	
		N (%)	N (%)	
Gender	Male (n=153)	119 (77.8)	34 (22.2)	0.029*
	Female (n=208)	140 (67.3)	68 (32.7)	
Age in years	Mean±SD	50.8±16.8	52.1±17.2	0.516^†^
Marital status	Single (n=74)	53 (71.6)	21 (28.4)	0.342*
	Married (n=237)	173 (73.0)	64 (27.0)	
	Divorced (n=12)	10 (83.3)	2 (16.7)	
	Widowed (n=38)	23 (60.5)	15 (39.5)	
Employment status	Unemployed (n=187)	128 (68.4)	59 (31.6)	0.407*
	Employed (n=58)	46 (79.3)	12 (20.7)	
	Student (n=20)	14 (70.0)	6 (30.0)	
	Retired (n=96)	71 (74.0)	25 (26.0)	
Educational level	Illiterate (n=83)	53 (63.9)	30 (36.1)	0.259*
	Primary-intermediate schools (n=95)	72 (75.8)	23 (24.2)	
	Secondary school (n=79)	60 (75.9)	19 (24.1)	
	University/postgraduate (n=104)	74 (71.2)	30 (28.8)	

Diabetes-Related Factors

Patients with a history of diabetic complications were more likely than their counterparts to use CAM (32% vs. 22.5%). However, this difference was borderline insignificant, p=0.052. Ex-smokers were more likely than never and current smokers to use CAM (40.9% vs. 28.3% and 15.6%, respectively), p=0.029. Other studied factors (type of diabetes, duration of diabetes, diabetic medications, co-morbidities and Glycated hemoglobin) are given in Table 5.

**Table 5:** Diabetes-related factors associated with complementary and alternative medicine intake among diabetic patients *Chi-square test HbA1c: Glycated hemoglobin

		Complementary and alternative medicine use		p-value*
		No N=259 N (%)	Yes N=102 N (%)	
Type of diabetes	Type 1 (n=102)	71 (69.6)	31 (30.4)	0.571
	Type 2 (n=259)	188 (72.6)	71 (27.4)	
Duration of diabetes in years	≤5 (n=79)	57 (72.2)	22 (27.8)	0.947
	6-10 (n=78)	55 (70.4)	23 (29.5)	
	11-15 (n=63)	47 (74.6)	16 (25.4)	
	>15 (n=141)	100 (70.9)	41 (29.1)	
Diabetic medications	Oral hypoglycemics (n=89)	62 (69.7)	27 (30.3)	0.429
	Injectable hypoglycemic agents (n=13)	7 (53.8)	6 (46.2)	
	Insulin (n=141)	105 (74.5)	36 (25.5)	
	Combinations (n=118)	85 (72.0)	33 (28.0)	
Diabetic complications	No (n=142)	110 (77.5)	32 (22.5)	0.052
	Yes (n=219)	149 (68.0)	70 (32.0)	
Comorbidities	No (n=108)	74 (68.5)	34 (31.5)	0.374
	Yes (n=253)	185 (73.1)	68 (26.9)	
HbA1c% (n=351)	≤7 (n=116)	87 (75.0)	29 (25.0)	0.348
	>7 (n=235)	165 (70.2)	70 (29.8)	
Smoking status	Non-smoker (n=272)	195 (71.7)	77 (28.3)	0.029
	Current smoker (n=45)	38 (84.4)	7 (15.6)	
	Ex-smoker (n=44)	26 (59.1)	18 (40.9)	

Multivariate logistic regression analysis revealed that females were at doubled likelihood to use CAM compared to males (Adjusted odds ratio “AOR” = 2.40; 95% confidence interval “CI”: 1.27-4.52, p=0.007). Compared to never-smokers, ex-smokers were more likely to use CAM (AOR=2.87; 95% CI: 1.28-6.43, p=0.010). A history of diabetic complications was not significantly associated with CAM use (Table 6).

**Table 6:** Predictors of using complementary and alternative medicine among diabetic patients: Results of univariate and multivariate logistic regression analysis a: Reference category

		Unadjusted odds ratio	95% confidence interval	Adjusted odds ratio	95% confidence interval	p-value
Gender	Male^a^	1.0		1.0		
	Female	1.70	1.05-2.74	2.40	1.27-4.52	0.007
Smoking status	Non-smoker^a^	1.0		1.0		
	Current smoker	0.47	0.20-1.08	0.86	0.33-2.27	0.764
	Ex-smoker	1.75	0.91-3.38	2.87	1.28-6.43	0.010
Comorbidities	No^a^	1.0		1.0		
	Yes	1.25	0.76-2.04	1.60	0.97-2.63	0.068

## Discussion

This study emphasizes the prevalence, determinants and pattern of complementary and alternative medicine use among diabetic patients which could increase the importance of taking history regarding CAM use that might affect the management plan.

The use of CAM to treat several diseases in Saudi Arabia is something hardly related to the culture and traditional values of the country [8]. The prevalence of using CAM among diabetic patients in this study was 28.3%, while the prevalence of using CAM for treating diabetes in the last 12 months was 22.4%. This figure is lower than that reported in a recent systematic review and meta-analysis that included 38 studies, which revealed a pooled prevalence of CAM use as 51% [9]. Also, it is lower than figures reported from Qassim (45.2%) [10] and Riyadh (64%) [11] in Saudi Arabia as well as from Ethiopia (73.7%) [12], Nigeria (62.1%) [13], and Iran (75.3%) [14], however, it is comparable to figures reported in other Saudi studies carried out in Jazan (34%) [15], Taif (33.7%) [16], Riyadh (26%) [7], and Jeddah (30.5%) [17], as well as figures reported from Thailand (30.89%) [18] and the United States (26.2%) [19]. On the other hand, the figure reported in the present study was higher than that reported from Jordan (16.6%) [20]. In India, exclusive CAM use to treat diabetes was reported by 9% while exclusive modern medicine was reported by 61% and combined use was mentioned by 30% of patients [21]. In Oman, 42% of the diabetic patients used CAM to treat the disease [22]. Furthermore, in Eastern Mediterranean countries, the frequency of using CAM ranged from 9 to 88.4% [23]. The variations in the rate of CAM use among diabetic patients in the aforementioned studies could be partially explained by differences between countries and regions in the accessibility and availability of modern medicine as well as socio-cultural perceptions of CAM use among patients.

In the current study, the majority of patients who reported using CAM (97.1%) used herbs, mainly cinnamon (48.5%), fenugreek/helba (31.3%) and rosemary (20.2%). In a previous study carried out in Taif, all diabetic patients reported using bitter apple, 66.1% using cinnamon, 55.1% using ginger, 35.6% took fenugreek, and 21.2% reported using garlic as an only CAM [16]. In Riyadh, the most commonly used herbal supplements were black cumin (42%), followed by fenugreek (28%), myrrh (24%), frankincense (22%), cinnamon (15%), garlic (15%), and onion (15%) [7]. The use of medicinal herbs in almost all parts of Saudi Arabia is a traditional habit for hundreds of years as awareness about CAM use in the treatment of different illnesses is being passed from one generation to other and is widely accepted by all socio-economic levels, educational levels and in all geographical regions [15, 24]. Also in other studies carried out elsewhere, the commonest used CAM to manage diabetes was herbs [12, 23, 25].

Several studies indicated the antidiabetic activities of herbal medications [26], as they are rich in secondary metabolites such as terpenoids, flavonoids and coumarins that have shown hypoglycemic activity [27]. The antihyperglycemic effect of herbal medicines could be explained by many mechanisms including β-cell K+ channel blocking, glycogenesis stimulation, promotion of regeneration of the β-cells, enhanced insulin release, inhibition of gluconeogenesis and glycogenolysis, decreased insulin resistance, stimulation of glycolysis, preventing oxidative stress as well as inhibition of α-glucosidase and β-galactosidase [28].

The high utilization of herbal medicines by patients with diabetes could be attributed to their availability, tradition in their use to manage many illnesses, and easy accessibility without regulatory control [12].

The main source of information about CAM among the participants in this study was family and friends (64.7%), followed by media and advertisements (24.5%). Also in Jazan (Southern Saudi Arabia), media, family and friends were the main source of information about CAM [15]. This finding is an alarming one that shows a lack of proper CAM-related information and this needs proper intervention from the responsible higher authorities.

A history of improvement in blood sugar reading with CAM was reported by 61.8% of patients. In another similar study conducted in Riyadh, 51% of patients reported improvement in their blood sugar levels after CAM use [7].

The history of informing treating physicians about CAM was mentioned by only 11.8% of patients in the present study. This figure is comparable to those reported by Argáez-López et al. in Mexico [29]. However, a higher rate was reported in Iran (59.1%) [12]. In this regard, treating physicians should be informed about the use of CAM therapies by their diabetic patients as this could change their plan of management. However, in the present study, the majority of patients reported no changes.

The present study revealed that female patients were more likely than males to use CAM. However, other socio-demographic factors such as age, marital status, employment status and educational level did not affect the CAM usage among the patients. The same has been observed, with the exception of gender, in similar other Saudi studies [11, 15, 17]. Gender role in the CAM use among diabetic patients is inconclusive in the literature; some found no role [12], some reported male predominance (UAE) [25] while others, in accordance with our finding observed female predominance [16]. In Eastern Mediterranean countries members of the WHO, factors affecting the consumption of CAM were age, female gender, and duration of diabetes [23]. In Iran [12], patients’ age, residence, marital status, family history of diabetes, diabetic complications as well as duration of diabetes were significant determinants of CAM use. In Taif, female gender, family history, diabetic complications, and longer duration of diabetes were associated with the increased use of CAM [16]. In Riyadh, older age and employment status were predictors of CAM use in Saudi patients with diabetes [7].

In this study, ex-smokers were more likely to use CAM than current and never-smokers. We did not find similar findings in other studies to compare. This behavior aligns with the idea that individuals who successfully quit smoking may be more proactive in seeking alternative therapies to complement their conventional medical treatments, particularly in managing conditions like diabetes, which require ongoing lifestyle adjustments. This finding could be studied thoroughly in future researches.

In both bivariate and multivariate analyses, diabetic patients with complications were more likely, although not reaching significant levels (p=0.052), than their counterparts to use CAM. This near-significant finding was statistically significant in another study conducted in Taif [16]. Higher use of CAM among diabetic patients with complications was observed in studies carried out in Iran [12]. It is believed that diabetic patients turn to CAM more as the disease becomes difficult to control and complications appear [25]. Larger population studies would help clarify this difference.

Strengths and limitations

The strength of the present study is that it is one of the limited Saudi studies that investigated the rate of pattern of use of CAM among diabetic patients. Despite that, it has some important limitations that should be addressed. Its cross-sectional design allows only assessing association and not causality between independent factors and the outcome. Data were collected through interviewing of patients which could impact their responses. Finally, patients were recruited from a single healthcare facility which could affect the generalizability of findings over other settings.

## Conclusions

The use of CAM to treat diabetes is a relatively common practice among Saudi patients, particularly herbs like cinnamon, fenugreek/helba and rosemary. History of improvement of blood sugar reading with CAM was reported by most of them while history of informing treating physicians about CAM was reported by minority of patients. Females and ex-smokers were more likely to use CAM than their peers. Based on the study’s findings, we recommend integrating CAM discussions into routine diabetes care or developing patient education materials in collaboration with CAM practitioners about the safety and proper use of CAM products. Diabetic patients should be encouraged to inform their treating physicians about the use of CAM products through creating trustworthy relationships between both. Finally, further longitudinal study is needed including diabetic patients from different sectors and different regions in order to have more comprehensive results.
